# Truncal necrobiosis lipoidica diabeticorum: A first case report

**DOI:** 10.1016/j.ijscr.2020.11.009

**Published:** 2020-11-08

**Authors:** Maram Alkhatieb, Hatan Mortada

**Affiliations:** a Division of General Surgery, Department of Surgery, Faculty of Medicine, King Abdulaziz University, Jeddah, Saudi Arabia; b Division of Plastic Surgery, Department of Surgery, College of Medicine, King Saud University, Riyadh, Saudi Arabia

**Keywords:** NL, necrobiosis lipoidica, DM, diabetes type, Necrobiosis lipoidica diabeticorum, Truncal, Diabetes mellitus, Skin lesion, Granulomatous dermatitis, Case report

## Abstract

**Introduction:**

Necrobiosis lipoidica (NL) is a known chronic rare granulomatous dermatitis. NL typically occurs in the lower extremity, there have only been previously reported cases of NL on the face, scalp, forearms, and dorsum of the hands. However, truncal NL is not a typical location.

**Presentation of case:**

A 67-year-old man known to have diabetes type 2, presented with a 3-year history of painful and itchy skin lesions on the right lateral chest wall. On clinical examination, he had a red discolored patch with superficial capillaries and yellow discoloration at the center. Based on the histological examination, necrobiosis lipoidica was confirmed. Initial treatment with clobetasol propionate, tacrolimus, and topical corticosteroid cream was unsuccessful, and further NL lesions developed on the lower limb.

**Discussion:**

NL rash is characterized by its location in the lower limbs and rarely involves fingers, hands, scalp, or face. It is noteworthy that the patient presented with NL skin lesions bon the trunk, which is outside the usual presentation. The prevalence of NL ranges from 0.3% to 1.2% among patients with diabetes mellitus. The treatment of NL is usually difficult. Initial management includes smoking cessation and proper diabetes control. In addition, intralesional and topical corticosteroids might be effective. In this case report, there was no improvement in NL despite the use of topical corticosteroids.

**Conclusion:**

Immediate diagnosis and treatment of NL may prevent disease progression; therefore, the diagnosis of NL should be considered, even in an unusual location.

## Highlights

• Necrobiosis lipoidica is a rare chronic dermatitis found in diabetic patients.
• These skin lesions affect 0.3–1.2% of patients with diabetes mellitus.
• Our patient developed necrobiosis lipoidica on the trunk, an unusual location.
• Treatment of necrobiosis lipoidica is difficult, related to good glycemic control.

## 1 Introduction

Necrobiosis lipoidica (NL) is a known chronic rare granulomatous dermatitis. First defined by Oppenheim in 1929, it typically occurs in the lower limb and affects around 0.3–1.2% of patients with diabetes mellitus (DM), mostly type 1. [1] According to a large series study by Muller and Winkelmann, more than half of the patients (65%) with NL had diabetes at presentation [2]. In contrast, another study showed that only 11% of diabetic patients had NL at presentation. Although lacking full concordance, NL remains an important skin lesion marker of DM [3]. On clinical examination, NL is characterized by sclerodermiform inflammatory plaques on the shins. The hallmark in the histopathological examination of the NL is the linear tiers of palisading necrobiotic granulomas aligned parallel to the skin surface, usually centered in the lower dermis [4]. Although NL typically occurs in the lower extremity, there have also been previously published case reports of NL on the face, scalp, forearms, and dorsum of the hands [5]. However, truncal NL is not a typical location. Based on a comprehensive literature search, this report is the first documented case of NL occurring on the trunk in an adult. Thus, we present a case of a 67-year-old man who developed NL lesions on the trunk who came to our outpatient clinic in a university hospital. This work has been reported in line with the SCARE (Surgical Case Report) Guidelines [6].

## 2 Case presentation

A 67-year-old Saudi man presented to the outpatient clinic of our university hospital with a 3-year history of painful and itchy skin lesions on the right lateral chest wall posterior to the axillary line. The lesions were constant in size and character. For 22 years, he had a history of type 2 diabetes mellitus, hypertension, and dyslipidemia. Diabetes was controlled using oral hypoglycemia agents and insulin. He was a smoker. Within the last 4 years, he also suffered from angina and renal impairment (secondary to post-contrast nephropathy) and has received treatment for gout the past year. At presentation, his latest fasting blood sugar was 5.2 mmol/L (normal range: 4.0–5.9 mmol/L) and glycosylated hemoglobin was 6.3% (normal range: 4.6%–6.5%). Apart from the above conditions, he was otherwise well. He has no history suggestive of any autoimmune disease. There was no history of trauma, medicine intake, or allergy to drugs or environmental agents. There was no family history of diabetes.

**Fig. 1:**
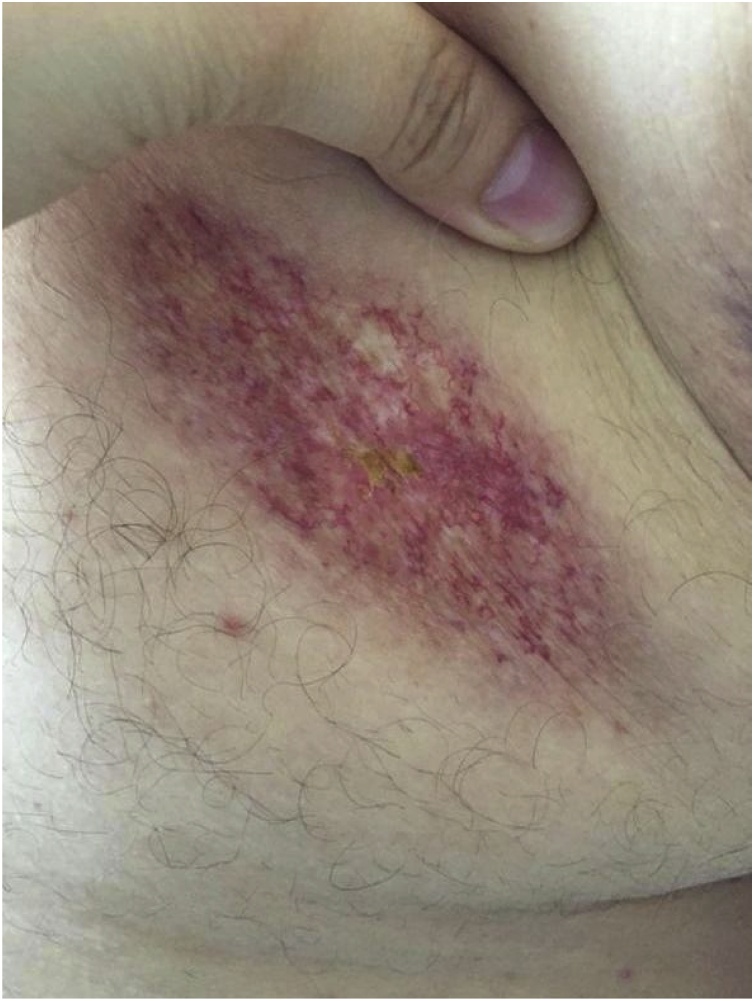
Clinical photograph of necrobiosis lipoidica on the trunk, showing darkly pigmented papules, plaques, a large annular lesion with hypopigmented atrophic center and prominent telangiectasia.

On clinical examination, he weighed 128 kg and his height was 180 cm (body mass index 39.5). On the right lateral chest wall posterior to axillary line, he had a red discolored patch with superficial capillaries and yellow discoloration at the center measuring approximately 2cm × 1cm, which remained constant, without changes in size or character (Fig. 1). Examination of the rest of the skin and other systems was unremarkable.

**Fig. 2:**
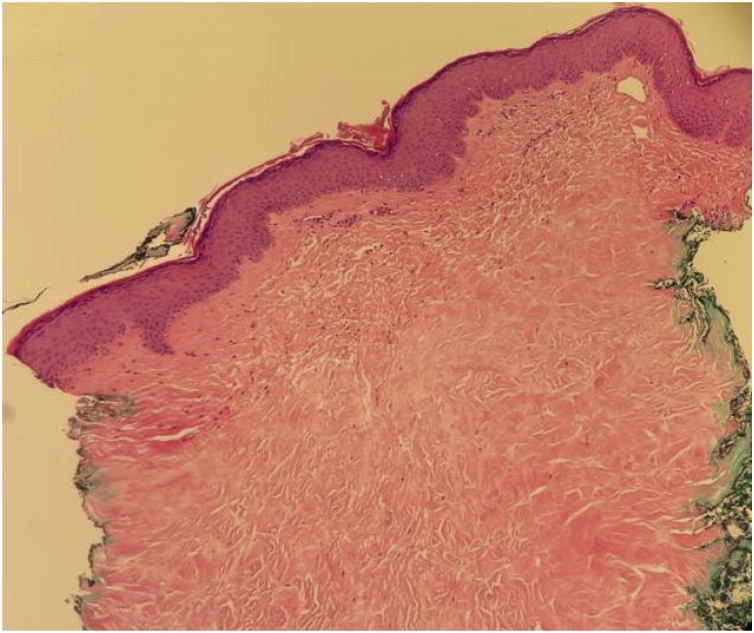
Histology of necrobiosis lipodica showing dense collagen material deposition and subcutaneous tissue sclerosis. The upper dermis and papillary dermis contained few inflammatory cells.

Histological examination of a 4-mm punch skin biopsy from the lesion edge showed subcutaneous tissue sclerosis and dense collagen material deposition. The upper dermis and papillary dermis contained few inflammatory cells (Fig. 2). The epidermis was unremarkable. Based on the appearance, necrobiosis lipoidica was confirmed without malignancy, and the appearance was not consistent with necrobiotic xanthogranuloma or sarcoidosis.

**Fig. 3:**
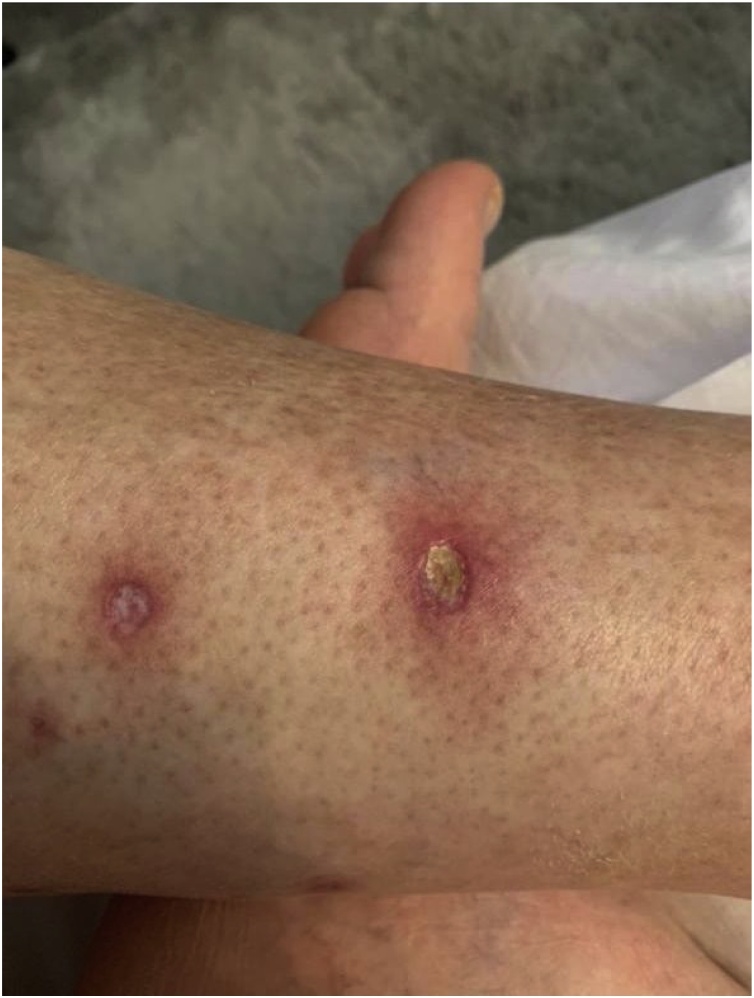
Necrobiosis lipoidica developed on the lower limb.

Initial treatment with clobetasol propionate, tacrolimus, and topical corticosteroid cream was unsuccessful, the patient is still being followed up and further NL lesions developed on the lower limb (Fig. 3). Smoking cessation was encouraged.

## 3 Discussion

Necrobiosis lipoidica is a granulomatous, distinctive dermatosis histopathologically characterized by collagen dermal necrosis, surrounded by palisading histocytes [7]. NL rash is characterized by its location in the lower limbs and rarely involves fingers, hands, scalp, or face [8]. It is noteworthy that the patient presented with NL skin lesions on the trunk, which is outside the usual presentation.

Typically, NL starts with skin lesions appearing as circular erythematous papules that then evolve to multiple, well demarcated, shiny, atrophic, brown to yellow telangiectasia with bilateral plaques. NL is generally asymptomatic unless it is ulcerated [1]. NL is more common in females than in males [8]. It usually appears during young and middle adulthood [9], although there are a few studies that document cases in childhood [10]. The prevalence of NL ranges from 0.3% to 1.2% among patients with diabetes mellitus [1], of which two-thirds have type 1 diabetes.

The pathophysiology of NL is idiopathic. One theory suggests that NL is one of the possible manifestations of microangiopathy, owing to its clear association with diabetes. Verrotti et al. have reported that diabetic patients with NL might be an alarming manifestation of retinopathy and nephropathy [11]. In contrast, poorly controlled glucose is associated with the development and progression of NL lesions [12]. Hence, well-controlled glycemic measures play a major role in preventing NL or even improving skin lesions, if present [12].

The treatment of NL is usually difficult. Initial management includes smoking cessation and proper diabetes control. In addition, intralesional and topical corticosteroids might be effective [13]. In this case report, there was no improvement in NL despite the use of topical corticosteroids. Precautions must be taken, however, as using systemic medications such as corticosteroids or azathioprine could enhance malignant transformation [14]. Previous studies have reported the occurrence of squamous cell carcinomas in areas of NL [14].

## 4 Conclusion

In conclusion, we report a rare case of NL of the trunk in a 67-year-old diabetic man. Immediate diagnosis and treatment may slow or prevent disease progression; therefore, the diagnosis of NL should be considered when presented with skin lesions on the trunk of a diabetic patient. Even in this unusual location, its association with diabetes must be ruled out.

## Declaration of Competing Interest

None.

## Funding

None.

## Ethical approval

Our institution does not require ethical approval for reporting case reports.

## Consent

Written informed consent was obtained from the patient for publication of this case report and accompanying images. A copy of the written consent is available for review by the Editor-in-Chief of this journal on request.

## Author contribution

Maram Alkhateib: Study concept or design, data collection, writing the paper.

Hatan Mortada: Data collection, interpreation, writing the paper

## Registration of research studies

N/A.

## Guarantor

Maram Alkhatieb.

## Provenance and peer review

Not commissioned, externally peer-reviewed.
